# COPSOQ III in Germany: validation of a standard instrument to measure psychosocial factors at work

**DOI:** 10.1186/s12995-021-00331-1

**Published:** 2021-11-16

**Authors:** Hans-Joachim Lincke, Martin Vomstein, Alexandra Lindner, Inga Nolle, Nicola Häberle, Ariane Haug, Matthias Nübling

**Affiliations:** FFAW Freiburger Forschungsstelle für Arbeitswissenschaften GmbH, FFAW Freiburg research centre for occupational sciences, Bertoldstr. 63, 79098 Freiburg, Germany

**Keywords:** COPSOQ, Psychosocial stress, Risk assessment, Work factors, Validation

## Abstract

**Background:**

Over the last almost 20 years COPSOQ (Copenhagen Psychosocial Questionnaire) has become a well-established instrument to measure psychosocial stress at work. In Germany, a first validated version of COPSOQ was introduced in 2005. After the COPSOQ international network took over responsibility for the development of COPSOQ, a new version was published in 2019 (COPSOQ III). The German version of this questionnaire is now to be validated.

**Methods:**

Measurement qualities of German COPSOQ III are explored in adherence to the to the usual requirements of a validation study as defined by DIN EN ISO 10075-3. A sample of observations from more than 250,000 participants surveyed with the COPSOQ in Germany is used for univariate and multivariate statistical analysis.

**Results:**

With its 84 items the German COPSOQ III includes all psychosocial work factors that are internationally obligatory and is still compatible with almost 70% of the content in the 2005 German version. Typical psychometric properties of the questionnaire (e. g., validity and reliability) are either good or very good for most of the 84 items and 31 scales. Beyond basic results, congruences with widely used theoretical approaches like the Demand-Control(−Support) model or the Job Demands-Resources model are generally satisfactory.

**Conclusions:**

With the launch of COPSOQ III in Germany, new workplace psychosocial aspects could be explored. Like the preceding version, the questionnaire is a highly useful instrument for research as well as for risk assessment in enterprises. COSPQO III covers a multitude of theoretical approaches and gives comprehensive information on psychosocial working conditions to deduce actions for their improvement.

**Supplementary Information:**

The online version contains supplementary material available at 10.1186/s12995-021-00331-1.

## Abstrakt

**Hintergrund:**

Seit fast 20 Jahren ist der COPSOQ (Copenhagen Psychosocial Questionnaire) ein weltweit etabliertes Instrument zur Messung psychischer Belastungen bei der Arbeit. In Deutschland wurde eine erste validierte Version des COPSOQ im Jahr 2005 eingeführt. Nachdem das internationale COPSOQ-Netzwerk die Verantwortung für die Entwicklung des COPSOQ übernommen hatte, wurde 2019 eine neue Konzeption veröffentlicht (COPSOQ III). Die deutsche Version dieses Fragebogens soll nun validiert werden.

**Methoden:**

Die Messqualitäten des deutschen COPSOQ III werden gemäß den üblichen Anforderungen an eine Validierungsstudie untersucht, wie sie mit DIN EN ISO 10075-3 definiert werden. Eine Stichprobe aus Beobachtungen von mehr als 250,000 Teilnehmenden an COPSOQ-Befragungen steht für univariate und multivariate statistische Analysen zur Verfügung.

**Ergebnisse:**

Neben der Einbeziehung sämtlicher psychosozialer Belastungsfaktoren gemäß der internationalen Festlegung, ist der deutsche COPSOQ III mit seinen 84 Items noch zu fast 70% inhaltlich mit der deutschen Version von 2005 kompatibel. Die psychometrischen Eigenschaften des Fragebogens (z. B. Validität und Reliabilität) sind für die meisten der 84 Items und 31 Skalen gut bis sehr gut. Jenseits der grundlegenden Ergebnisse bestehen insgesamt zufriedenstellende Übereinstimmungen mit weit verbreiteten theoretischen Ansätzen wie dem Demand-Control(−Support)-Modell oder dem Job Demand-Resources-Modell.

**Schlussfolgerungen:**

Mit der Veröffentlichung des COPSOQ III in Deutschland lassen sich neue psychische Faktoren untersuchen. Wie bei der vorangehenden Version handelt es sich um ein Instrument von hohem Nutzen sowohl für die Wissenschaft als auch für die Gefährdungsbeurteilung in Unternehmen. Der COPSOQ III deckte eine Vielzahl theoretischer Ansätze ab liefert umfassende Informationen über Arbeitsbedingungen, aus denen sich Verbesserungsmaßnahmen ableiten lassen.

## Background

In the last 20 years the Copenhagen Psychosocial Questionnaire (COPSOQ) has become a popular instrument for research and risk assessment of workplace psychosocial conditions all over the world [1]. Two qualities need to be demonstrated for an instrument to achieve such popularity: On one hand it must be time indifferent, as deep insights usually need to be observed at more than one event. On the other hand, it should be sensitive to new trends to keep pace with a rapidly changing world. However, according to the COPSOQ international network (www.copsoq-network.org), a good questionnaire should not only care about time but also space. With its new guidelines on COPSOQ III the network wants to achieve comparability of data on an international level and a reflection of local cultural contexts at the same time. Following the guidelines, any questionnaire of this new type must contain a fixed set of common, so-called “core questions” and also an indefinite number of questions of national relevance [1]. Hence, like other national versions of COPSOQ III the German questionnaire is not a ready-to-use-collection of question, defined somewhere else and now translated into German. It is better understood as a purposeful combination of the internationally mandatory core questions with questions that are predominantly used in Germany.

Historically, a first COPSOQ questionnaire was validated and published in Germany by Nübling et al. in 2005 [2, 3]. This questionnaire was based on the initial Danish version developed by Kristensen et al. what is nowadays called “COPSOQ I” [4]. While COPSOQ I had a promising start in Germany, Kristensen and his colleagues soon launched COPSOQ II based on their experiences with the initial questionnaire [5]. Researchers from some countries like France [6], Spain [7], Sweden [8], and Hungary [9] switched to COPSOQ II as it offered e. g. several levels of complexity (short, middle, long version), while others went on with the initial version or, like in Germany, just picked out some constructs of COPSOQ II. In Germany, only four items concerning organisational trust and justice were selected from COPSOQ II in 2010. At the same time, scales on presenteeism (working while being ill) and physical demands with non-COPSOQ origins were added as well.

After some years, it became more and more clear that the alternation between first and second versions, different levels of complexity, and national editions would soon dissolve the reputation of “COPSOQ” as a well-known label for a well-functioning set of scales and items. For this reason, the steering committee of the COPSOQ international network [10], assumed responsibility for the future development of the questionnaire and in 2013 invited all network members to contribute to the elaboration of a new COPSOQ standard. In 2017, COPSOQ III was released and after some testing in six countries, and minor changes were published in 2019 [1].

In relation to its guidelines, COPSOQ III might be seen rather as a manual on how to build a questionnaire rather than a ready-to-use-questionnaire. In Germany, the Freiburg research centre for occupational sciences (FFAW) is the national group for COPSOQ-related issues. The FFAW viewed proceeding to COPSOQ III as adjusting the already existing questionnaire to a new standard. First, the new COPSOQ III’s definition of 32 mandatory core items meant that the new German COPSOQ III had to add the following items: two items concerning work pace, one item dealing with emotional demands, and two items regarding work-privacy conflicts. Second, it meant an occasion to view past items critically. Scales on Satisfaction with Life (had proved to be too intrusive), Cognitive Stress Symptoms (were highly redundant with Burnout Symptoms), and a few single items had proved inefficient, and were excluded from further use. Third, proceeding to COPSOQ III meant the chance to add some “fresh” content: one item on recognition by management, one item on satisfaction with salary, one item on the inability to relax, two items on insecurity over working conditions, two items concerning dissolution of work and private life, and a scale with three items on work engagement were introduced as country specific items of COPSOQ III in Germany.

**Fig. 1:**
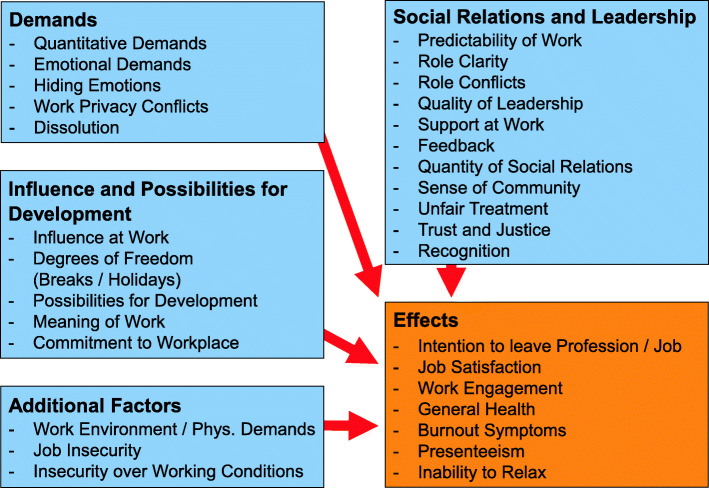
Scales of the German COPSOQ III questionnaire

Additional file 1 shows how the national and international perspectives correspond by presenting questions (items) and factors (scales) of the German COPSOQ III, their origins, the years they had been used for the first time in a German COPSOQ version, and the current names as variables in the international and national context. Obviously, many items and scales of COPSOQ III had been already part of the COPSOQ questionnaire since 2005. In numbers, 58 items out of 84 items (almost 70%) are identical. This ensures a high content continuity and potential comparability of data collected over the years. Figure 1 gives an impression of the COPSOQ III scales included in Germany, organised in a model of cause and effect [11], with main dimensions sorted a priori to correspond closely with the Demand-Control-Support model [12, 13]. This set of scales will be explored empirically.

## Methods

There are some common criteria to describe the qualities of a questionnaire. DIN EN ISO 10075-3 [14] is a systematic compilation of criteria that we used as a framework to analyse the qualities of German COPSOQ III. Some of the criteria cannot be assessed with statistical analysis, as the desirable data do not exist or cannot be gained with acceptable efforts. Objectivity (comprising consistency, stability, inter-rater-reliability, test-retest-reliability), as well as external validity and usability are examples of criteria that cannot be met with statistical analyses. Other criteria that can be met with statistical analysis of empirical data are the reliability and homogeneity of scales (Cronbach’s α, ICC), their sensitivity, variance, and characteristics of distribution (descriptive measures, floor / ceiling effects), internal validity and distinctiveness (Pearson’s r), and diagnosticity (analysis of variance). Multivariate models are added based on explorative factor analysis (EFA) and linear regression.

Due to its activities in scientific research and risk assessment in companies since 2005, FFAW was able to collect a large amount of empirical data. In March 2020, the COVID-19 pandemic began influencing working conditions in Germany. There are first impressions related to the European Union (EU) [15] and even results based on a COPSOQ survey in Spain [16]. But as not all influences can yet be foreseen, the sample for validation of COPSOQ III was drawn strictly from surveys conducted in the pre-pandemic time period (January 2015 – February 2020).

The sample comprises the anonymised observations of 257,236 participants from 966 surveys on risk assessment in companies throughout Germany. These surveys were conducted in accordance with General Data Protection Regulation (GDPR) of the EU. Usually, every employee of a company was invited to fill in the questionnaire, that was made available via internet and accessible either by a generic or individual password. Average participation rate was 61.4% (median 62.2%). Unfortunately, no information on non-responders can be gained this way.

Any information on participants’ occupations is classified according to the most complex level of the official German Classification of Occupations (KldB 2010) [17]. Thus, the sample comprises 971 occupational types (job titles) at the most detailed level of 5 digits, which can be aggregated to 49 occupational groups on the 3-digit level. At the 3-digit level, the sample can be weighted using official numbers published by the Federal Statistical Office (Destatis) as a reference to reflect the real distribution in Germany [18]. For the more comprehensive analysis presented here, the original occupational groups were condensed to the 1-digit level that in the official terminology defines the more generalised “occupational areas”.

All of the questionnaire items have a Likert-type scale and values for the coding of responses range from 0 (minimum value, e. g. “does not apply at all”) to 100 points (maximum value, e. g. “fully applies”). Except for the question on general health which uses a ten-interval answer scale, there are always five possible answers with values corresponding to 0, 25, 50, 75 and 100 points. Mean values of scales are calculated if at least half of the affiliated items are non-missing. In accordance with the coding of answers, mean values of items and scales always range between 0 and 100 points.

## Results

### Non-statistical checks and sample characteristics

It is very important to know to which extent a questionnaire is really measuring what it is supposed to measure. Content validity is not necessarily a matter of statistics, but of the certitude that items and scales really cover their subjects and, of course, that the selection of subjects is wise. The selection of the 32 international core items ensuring completeness and relevance of subjects, was obviously the international network’s task. The steering committee and all network members discussed the substantial and statistical values of all items and scales with this goal [1]: In a first Delphi-like phase beginning in 2013, the steering committee asked network members and external researchers to make propositions on favoured subjects plus related scales and items, to comment the emerging collection, to make new propositions, etc.. A preliminary questionnaire was then introduced at the network conference in Paris in 2015. This phase was followed by empirical testing until 2017. Last decisions and agreements were made, and finally presented in 2019 as COPSOQ III at a conference in Santiago de Chile.

This process makes clear that content validity of COPSOQ III is predominantly assured by literature and expert knowledge, not by studies on potential survey participants. It is the international network’s principle that core items and any national additions shall be based on tried and tested, and thus already validated questions. Additional file 1 shows that for the German questionnaire this specification was followed. Thus, there was no need to check for consistency by comparing results of questions with any results of questions that have an identical content in the same survey.

The criterion of stability in terms of test-retest-reliability would usually mean to count how often a respondent would give the identical answers in a certain period of time. This check was excluded, first for practical reasons: It would have been time-consuming, expensive, and difficult to explain in a company setting that the participants should fill in the questionnaire many times in short intervals. But second, it seemed unnecessary, as almost all items and scales had been originally tested before their inclusion in earlier versions of the COPSOQ. Stability in terms of inter-rater-reliability was not checked, as this criterion simply does not apply to an instrument bound to self-observation.

The degree to which results are independent of the way the questionnaire is used can be defined as its objectivity. In this sense, the process of asking questions, giving answers, and data analysis is highly objective. First, the questionnaire is always filled in by the participants themselves – there is no interviewer who could influence their answers. Second, the surveys in all companies follow the same fixed scheme, and third, procedures to transform answers into statistical data are predefined and invariant.

We regard the criteria of usability as being met, since contact persons from companies where surveys were conducted consistently confirm that the surveys run smoothly. Privacy standards are according to the General Data Protection Regulation (GDPR) of the EU. The online version is easy to find and to access, the paper-pencil-version can be distributed and returned reliably per mail. In a text-field asking for practicability included in all German surveys, questions and answers are usually said to be simple and easy to understand. In the sample analysed, the average time to fill in the questionnaire online was 24 min (median 20 min). The average participation rate was 61.4% (median 62.2%). Unfortunately, there is no information available to distinguish between those who took part in the surveys and those who refused.

Table 1 gives an overview of the sample. From a socio-demographic perspective 48.3% of the participants in the sample were female and 51.7% male – a third response option was included starting in 2019 and therefore not yet offered in most of the included surveys, so these results are not displayed. These values are quite close to the official numbers for the working population Germany (46.6% female, 53.4% male) [18]. Concerning age, the group up to 24 years was the smallest one comprising 6.2%. The three groups with an age from 25 to 54 years ranged in size between 20 and 30%, while the group 55 years and older encompass 17.7%. In comparison with official numbers, the oldest age group is underrepresented by 5.8%, while all other age-groups showed smaller differences of 2 to 4%.

**Table 1:** Study sample: socio-demographic and occupational characteristics

Feature	Sample of COPSOQ-database 2015–2020 (***n*** = 257,236)			National statistics^b^
	Category	Frequency^a^	Percent^a^	Percent
Gender	male	129,181	51.7	53.4
	female	120,916	48.3	46.6
Age groups	up to 24	15,634	6.2	9.5
	25–34	57,022	22.5	20.2
	35–44	60,181	23.8	20.7
	45–54	75,552	29.9	26.2
	55 and more	44,702	17.7	23.5
Fixed-term contract	yes	20,034	13.0	8.9
	no	133,762	87.0	91.1
Full-time work	yes	175,088	73.9	70.2
	no	61,918	26.1	29.8
Working evening or night (≥ once/week)	yes	66,711	25.9	–
	no	84,428	32.8	–
Working weekend or holiday (≥ once/month)	yes	59,812	44.1	–
	no	85,479	55.9	–
Supervisor position	yes	40,850	19.8	–
	no	165,470	80.2	–
Occupational areas (KldB2010)	Agriculture, forestry, farming, and gardening	5372	2.1	–
	Production of raw materials and goods, and manufacturing	49,667	19.3	–
	Construction, architecture, surveying and technical building services	15,648	6.1	–
	Natural sciences, geography and informatics	9717	3.8	–
	Traffic, logistics, safety and security	33,423	13.0	–
	Commercial services, trading, sales, the hotel business and tourism	31,389	12.2	–
	Business organisation, accounting, law and administration	53,859	20.9	–
	Health care, the social sector, teaching and education	50,245	19.5	–
	Philology, literature, humanities, social sciences, economics, media, art, culture, and design	7916	3.1	–

^a^Difference to total number resp. 100% is caused either by question not asked or “no answer”, and in case of gender also “other”

^b^ Statistisches Bundesamt [18]; numbers for fixed-term and full-time work in official statistics without civil servants and trainees

A view on work contracts and working hours indicated that with 87.0% a vast majority of participants had permanent contracts and 73.9% were working full time. These rates are quite similar to the official rates of 91.1 and 70.2%. Working in the evening or at night was typical for 26.1% of the participants, and 44.1% worked on bank holidays or weekends. A view of the hierarchical position showed that 19.8% worked as supervisors. Regarding occupational areas according to KldB 2010, “Business organisation, accounting, law and administration” was the largest sector with 20.9%. This is probably because it is aggregating administrative work in public sector, as well as in companies. Almost of the same size are the sectors “Health care, the social sector, teaching and education” with 19.5% and “Production of raw materials and goods, and manufacturing” with 19.3%. The latter represents Germany’s industrial tradition. In accordance, “Agriculture, forestry, farming, and gardening” played a minor role with 2.1%.

### Descriptive analysis, correlations, and explorative factor analysis of scales

All 31 scales of the questionnaire are presented in Table 2. For each scale mean, standard deviation, and fractions with ceiling, floor, and missing values were calculated to check for sensitivity and variation. Covering the years 2015–2020, around 250,000 cases were available to analyse 25 of the 31 scales. The other 6 scales were integrated step-by-step until 2017, therefore the case numbers were necessarily lower. In a complete-case perspective, meaning to count only cases with no missing value, the total number of cases was 134,896. In a single scale perspective, the average rate of missing values was 3.6% with a range of 0.7–8.4%, while 22 out of 31 scales showed less than 5% missing values.

**Table 2:** Descriptive statistics and reliability of scales

Scale	Positive value	No. of items	N	Cronbach‘s α	Intra-class-correlation ICC	Scale mean	Std. deviation	Floor effect in %	Ceiling effect in %	Missing values in %
Quantitative Demands	low	5	253,437	0.81	0.46	55.4	19.0	0.4	0.9	1.5
Emotional Demands	low	2	254,517	0.74	0.58	47.7	27.9	9.8	6.6	1.1
Hiding Emotions	low	2	254,241	0.80	0.66	44.4	26.4	11.1	4.2	1.2
Work Privacy Conflicts	low	4	243,089	0.92	0.74	39.0	28.0	13.4	3.7	5.5
Dissolution	low	2	151,356	0.60	0.43	32.9	26.1	21.0	1.9	6.1
Influence at Work	high	3	254,551	0.75	0.51	43.7	22.9	5.4	1.0	1.0
Degrees of Freedom (Breaks / Holidays)	high	2	248,582	0.53	0.36	63.1	25.3	3.1	9.7	3.4
Possibilities for Development	high	3	255,236	0.78	0.54	63.1	22.0	1.3	5.8	0.8
Meaning of Work	high	2	255,425	0.85	0.74	74.4	21.5	0.9	25.8	0.7
Commitment to Workplace	high	2	255,276	0.79	0.65	59.2	25.7	3.3	11.9	0.8
Predictability of Work	high	2	253,035	0.76	0.61	52.3	22.0	2.7	3.3	1.6
Role Clarity	high	3	252,654	0.81	0.59	71.3	18.6	0.4	10.6	1.8
Role Conflicts	low	3	251,992	0.82	0.60	45.0	22.7	4.6	2.0	2.0
Quality of Leadership	high	4	248,729	0.91	0.71	52.9	25.4	4.3	4.5	3.3
Support at Work	high	4	252,017	0.82	0.54	69.4	21.2	0.3	11.6	2.0
Feedback	high	2	251,768	0.58	0.41	44.1	22.4	5.5	1.9	2.1
Quantity of Social Relations	high	1	249,808	–	–	57.4	28.1	7.9	14.8	2.9
Sense of Community	high	2	251,385	0.84	0.72	77.0	18.6	0.5	23.9	2.3
Unfair Treatment	low	1	249,487	–	–	21.3	25.0	48.2	1.2	3.0
Trust and Justice	high	4	242,664	0.81	0.52	60.5	18.5	0.6	2.6	5.7
Recognition	high	1	148,435	–	–	48.5	28.3	12.9	8.0	7.9
Work Environment / Phys. Demands	low	6	172,913	0.84	0.47	34.6	24.0	6.6	0.4	6.5
Job Insecurity	low	3	246,112	0.74	0.48	28.2	24.0	21.7	1.2	4.3
Insecurity over Working Conditions	low	3	147,998	0.77	0.52	28.6	24.8	20.5	1.7	8.2
Intention to leave Profession / Job	low	2	247,774	0.80	0.67	20.0	23.3	24.5	0.8	3.7
Job Satisfaction	high	7	248,837	0.82	0.40	63.1	16.9	0.1	1.6	3.3
Work Engagement	high	3	148,136	0.86	0.67	63.4	19.9	0.5	5.8	8.1
General Health	high	1	246,704	–	–	69.8	19.8	0.3	6.1	4.1
Burnout Symptoms	low	3	249,133	0.84	0.64	48.5	21.3	2.6	1.2	3.1
Presenteeism	low	1	241,957	–	–	41.9	26.1	14.7	3.4	5.9
Inability to Relax	low	1	147,739	–	–	45.8	28.4	13.4	6.6	8.4

The mean values of the German COPSOQ III version’s scales varied from 20.0 points for “Intention to leave Profession / Job” to 77.0 points for “Sense of Community”. The standard deviations of all scales reached from a minimum of 16.9 points to a maximum of 28.4 points. These values cannot be interpreted in a normative way. Of course, it would be favourable for a company, for example, if few persons were intending to leave (low is positive), and many enjoy working with each other (high is positive). But COPSOQ guidelines are not fixated upon any cut-off values, however legitimated, to be “the true” values. Thus, the really important information is that even the lowest and the highest mean values are in a distance of at least 20 points from 0 / 100 as the extreme ends of the possible value range. Floor effects – here defined as the percentage of answers coded zero – ranged between 0.2–48.2%. There were 5 scales with 20% and more on this category (“Insecurity over Working Conditions”, “Dissolution”, “Job Insecurity”, “Intention to leave Profession / Job”, “Unfair Treatment”), while 16 scales had less than 5% answers to this extreme. Ceiling effects – defined as the percentage of answers coded 100 – ranged between 0.4–25.8%. There were 2 scales exceeding 20% (“Sense of Community”, “Meaning of Work”), while 18 scales showed less than 5% answers on this extreme category.

Cronbach’s α and the intraclass-coefficient ICC were calculated to assess reliability and homogeneity for multiitem scales. There is a broad consensus that a value of α ≥ 0.7 shows acceptable reliability, and a value for ICC ≥ 0.5 is an indicator for an acceptable degree of congruence. In total, 28 scales showed a good or even very good reliability in relation to Cronbach’s α and another 24 scales showed satisfying or even good ICC. There were only three scales with low α and also low ICC: “Dissolution”, “Degrees of Freedom”, and “Feedback”.

It is understood to be a sign of good psychometric quality, when the relations between items of the same scale are close (but not too close). However, it is the opposite way with relations between scales. It is important to know, to what degree different scales represent different work factors. In Additional file 2 the internal validity and distinctiveness of scales in terms of correlation coefficients (Pearson’s r) is presented. Usually if r is lower than 0.1 the correlation is said to be negligible. Values up to |0.29| are said to stand for a weak correlation, while up to |0.49|, and |0.5| and more are interpreted as moderate and strong correlations, respectively. In this sense, out of a total of 465 correlations, r was weak in 318 cases (68.4%), moderate in 125 cases (26.9%), and strong in 22 cases (4.7%) with 0.64 as the highest value.

The strong correlations among work factors and effects are not difficult to explain. “Burnout Symptoms” could e. g. be recognised as health aspects and are as such tied to “General Health”. High ratings on “Quantitative Demands” can e. g., often mean having to work overtime creating difficulties balancing work and free time, or in other words, “Work Privacy Conflicts”. By asking for typical aspects of leadership, “Trust and Justice”, “Recognition”, “Quality of Leadership”, “Support at Work”, and the “Predictability of Work” are linked with each other. With regard to content, the question of how conflicts are solved by a superior (item in “Quality of Leadership”) is e. g., closely related to the question if conflicts are resolved in a fair way by the management (part of “Trust and Justice”). The extent that a superior is good at work planning (item in “Quality of Leadership”) is e. g., in part a question if all information needed to do the work well is received (also a question in “Predictability of Work”).

Explorative factor analysis (EFA) is an appropriate means to check statistical relations for a multitude of scales. The Tables 3 and 4 show the results of two EFA (extraction method: principal component analysis; rotation method: varimax with Kaiser normalization; eigenvalue of at least 1 as criterion) treating work factors and effects separately in accordance with the generalised model of cause and effect [11]. In the tables all factor loadings lower than |0.4| are hidden for better readability.

**Table 3:** EFA on psychosocial work factors: rotated factor matrix

Psychosocial work factors^a^	Factor loadings^b^				
	1	2	3	4	5
Meaning of Work	0.75				
Commitment to Workplace	0.68				
Role Clarity	0.64				
Predictability of Work	0.62				
Trust and Justice	0.60		0.42		
Recognition	0.59				
Possibilities for Development	0.51				0.47
Emotional Demands		0.76			
Quantitative Demands		0.71			
Work Privacy Conflicts		0.71			
Hiding Emotions		0.64			
Dissolution		0.64			
Role Conflicts	−0.44	0.51			
Support at Work			0.74		
Sense of Community			0.68		
Feedback			0.63		
Quality of Leadership	0.52		0.58		
Unfair Treatment			−0.45	0.42	
Insecurity over Working Conditions				0.80	
Job Insecurity				0.73	
Work Environment / Phys. Demands				0.61	
Degrees of Freedom (Breaks / Holidays)					0.65
Quantity of Social Relations					0.61
Influence at Work	0.40				0.60

^a^ Eigenvalue ≥1, total variance explained 56.2%

^b^ Only loadings ≥ |0.40| are shown

**Table 4:** EFA on effects: rotated factor matrix

Effects^a^	Factor loading^b^	
	1	2
Work Engagement	0.85	
Job Satisfaction	0.78	
Intention to leave Profession / Job	−0.75	
Presenteeism		0.77
Inability to Relax		0.70
Burnout Symptoms	−0.46	0.68
General Health		−0.59

^a^ Eigenvalue ≥1, total variance explained 61.3%

^b^ Only loadings ≥ |0.40| are shown

In Tables 3 components were extracted out of the 24 psychosocial work factors with the sum of squared loadings explaining 56.2% of the total variance. In Table 4 it can be seen that out of 7 scales on effects, 2 components were extracted, covering 61.3% of the total variance. These results were satisfactory, as widespread rules of thumb claim that an acceptable model should explain at least half of the total variance and the proportion of scales to factors extracted should be no less than 3:1.

**Table 5:** Regression models on satisfaction and health effects

Dependent Scale	Total model fit (number of all significant predictors)	Model fit with top five^a^ predictors	Top five^a^ predictors	Standardised Coefficient (Beta)
Intention to leave Profession / Job	R^2^ = 0.35 (22 predictors)	R^2^ = 0.34	Commitment to Workplace	−0.28
			Work Privacy Conflicts	0.22
			Age groups	−0.14
			Unfair Treatment	0.11
			Meaning of Work	−0.13
			Role Conflicts	0.09
Job Satisfaction	R^2^ = 0.66 (24 predictors)	R^2^ = 0.60	Quality of Leadership	0.31
			Commitment to Workplace	0.24
			Trust and Justice	0.19
			Sense of Community	0.17
			Work Privacy Conflicts	−0.17
Work Engagement	R^2^ = 0.47 (24 predictors)	R^2^ = 0.44	Commitment to Workplace	0.31
			Meaning of Work	0.20
			Possibilities for Development	0.18
			Work Privacy Conflicts	−0.15
			Role Clarity	0.12
General Health	R^2^ = 0.23 (22 predictors)	R^2^ = 0.22	Work Privacy Conflicts	−0.21
			Support at Work	0.08
			Age groups	−0.16
			Insecurity over Working Conditions	−0.13
			Commitment to Workplace	0.12
			Unfair Treatment	−0.10
Burnout Symptoms	R^2^ = 0.41 (21 predictors)	R^2^ = 0.37	Work Privacy Conflicts	0.40
			Trust and Justice	−0.12
			Hiding Emotions	0.13
			Gender	0.12
			Unfair Treatment	0.11
			Job Insecurity	0.10
Presenteeism	R^2^ = 0.22 (22 predictors)	R^2^ = 0.19	Work Privacy Conflicts	0.15
			Insecurity over Working Conditions	0.19
			Unfair Treatment	0.14
			Quantitative Demands	0.10
			Gender	0.11
			Dissolution	0.10
Inability to Relax	R^2^ = 0.20 (23 predictors)	R^2^ = 0.19	Work Privacy Conflicts	0.22
			Dissolution	0.17
			Job Insecurity	0.08
			Quantitative Demands	0.11
			Unfair Treatment	0.08
			Age groups	0.08

^a^ Top predictors are the first five workplace factors getting into a model. If gender or age group are getting in among the first five, a sixth predictor is listed

In Table 3, the factors numbered 1–3 combined a larger number of scales than factors 4 and 5. Component 1 showed high loadings for “Meaning of Work”, “Commitment to Workplace”, “Possibilities for Development”, but also a weaker loading for “Influence at Work” and could therefore be called “Influence and Possibilities for Development” in terms of dimensions in Fig. 1. Factor 3 strongly connected “Support at Work”, “Sense of Community”, “Quality of Leadership”, and “Feedback”, and could represent the dimension of “Social Relations and Leadership”. Obviously, there is a certain fuzziness between component 1 and 3 as “Quality of Leadership” and “Trust and Justice” are loading on both components.

In this perspective the clear correspondence of factors 2 and 4 with Fig. 1 is to be highlighted. Component 2 combined “Demands” as “Emotional Demands”, “Work Privacy Conflicts”, “Quantitative Demands”, “Hiding Emotions”, and “Dissolution”, while component 4 represented the “Additional Factors” as there are “Insecurity over Working Conditions”, “Job Insecurity”, and “Work Environment / Physical Demands”. Factor 5 finally seemed to connect scales of different dimensions, belonging either to the dimensions of “Influence” or “Social Relations” in the a priori model.

A high degree of distinctiveness is found among the 7 scales of effects. In Table 4 it can be seen that all scales loaded high on one of the two explored factors. Factor 1 stands for (dis-)satisfaction with working conditions combining “Work Engagement”, “Job Satisfaction”, and the “Intention to leave Profession / Job”. Factor 2 indicated health status in relation to work with “Presenteeism”, “Inability to Relax”, “Burnout Symptoms”, and, with a weaker tie to work, “General Health”.

### Regression models und group analysis

How much incongruence psychological models and theories may ever show, they all draw a distinction between causes and effects which are in so far related to each other as the first will shape the latter. This general consideration is important for everyone wanting to influence satisfaction or health state by applying improvement or preventive strategies in workplaces. If a given situation can be understood as a reaction (effect) of a specific kind of working conditions (causes), this will help to identify effective starting points for intervention measures. This idea leads to a statistical analysis of relationships between scales by means of linear regression. Table 5 illustrates the results of 7 multiple linear regression models (variables included stepwise). The satisfaction and health scales are each defined as outcome variables to be predicted by the 24 work factors plus gender and age group as independent variables. Because of the large number of scales, the results are presented in a compressed manner. At first, the table sums up the variance explained (model fit, determination coefficient R^2^) by a model including all statistically significant independent variables (out of 24 workplace factors plus age group and gender). The most relevant, top five workplace factors (plus gender or age group if included in the model among the first five workplace factors) are shown. Further workplace factors are not shown, as statistical significance alone is not a helpful criterium because it will emerge for small effects due to the mere size of the sample.

First, “Job Satisfaction” (R^2^ = max. 0.60 which means 60% of its variance is explained by the model) was predicted much better than all other effects. Next was “Work Engagement” (R^2^ = max. 0.44), followed by “Burnout Symptoms” (R^2^ = max. 0.37) and the “Intention to leave Profession / Job” (R^2^ = max. 0.34) with moderate explained variances. “General Health” (R^2^ = max. 0.22), “Presenteeism” (R^2^ = max. 0.19) and the “Inability to Relax” (R^2^ = max. 0.19) had relatively low values.

There were 17 out of 24 possible scales included in at least one of the models as predictors. Gender and age group were included in some of them, too. The most frequent independent factors in the models were “Work Privacy Conflicts” and “Unfair Treatment”, playing their roles in 7 and 5 models, respectively. All other factors emerged in up to 4 of the models. Furthermore, the scale on “Work Privacy Conflicts” was on the first rank in all models of health. The models on satisfaction tended to depend on scales that form the dimension “Influence and Possibilities”, like “Commitment to Workplace” or “Meaning of Work”, and hardly on scales from “Additional Factors” or on gender or age group. Just the other way round, “Insecurity over Working Conditions” and “Job Insecurity”, gender, and age group frequently appear in models on health. The newly included scale on “Dissolution” contributed to predict the “Inability to Relax”, which is deemed plausible.

**Fig. 2:**
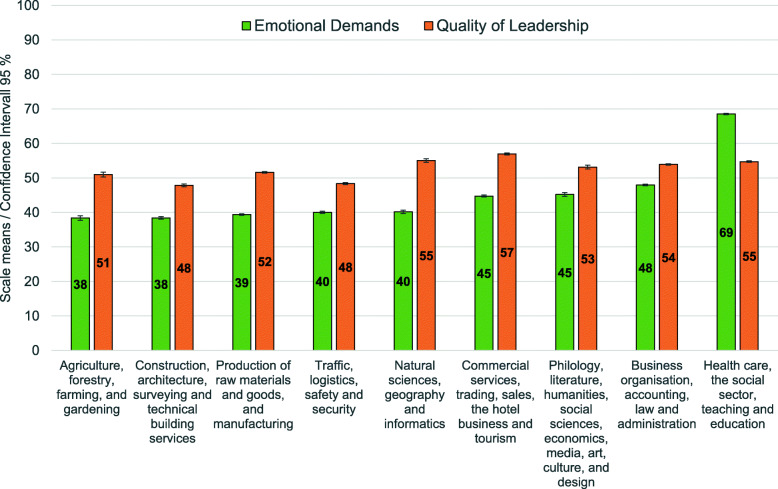
Emotional Demands vs. Quality of Leadership by occupational areas

The inference from causes to effects leads to the question if COPSOQ-scales can identify general types of working conditions. Diagnosticity / sensitivity was checked by examining the degree, to which well-known occupational groups with fixed activity patterns and thus “stress profiles” could be mapped. For this purpose, exemplary analyses of variance (ANOVA) were carried out for “Emotional Demands” and “Quality of Leadership”. The variance of these two scales shall be explained by occupational areas after KldB 2010 with eta^2^ as the measure of discrimination. Figure 2 depicts the mean values of the two scales for 9 occupational areas, sorted by the mean values for Emotional Demands.

As a matter of fact, different occupational groups face different “Emotional Demands” (total mean = 47.7; STD = 27.9). The rounded mean for working in agriculture, forestry, farming and gardening was 38 points. Commercial services, trading, sales, the hotel business and tourism is in the middle with 45 points, and health care, the social sector, teaching and education had to face 69 points. This is a span of 31 points between minimum and maximum values, and variance explained by occupational group is 15% (eta^2^ = 0.15), while for “Quality of Leadership” (total mean = 52.9; STD = 25.4), it is 1% (eta^2^ = 0.01). The range of mean values is narrow for this scale with a span of only 9 points between construction, architecture, surveying, and technical building services with 48 points (rounded) and traffic, logistics, safety and security, and commercial services, trading, sales, the hotel business and tourism with 57 points.

## Discussion

### Upgrading the first German COPSOQ version

The changes from the German COPSOQ version of 2005 to COPSOQ III can be seen as an adaption to the concept of an international core set of questions, as well as an adaption to changes in occupational theories and work environment. As changes are moderate, the high quality of COPSOQ surveys in Germany that was achieved during the last years is not put at risk.

The non-statistical qualities of German COPSOQ III, like content validity, objectivity and usability are pretty similar compared to the COPSOQ version of 2005. Both questionnaires rely on already tried and tested scales, and due to the newer version’s high congruence to the older version’s content, COPSOQ III can rely on the fact that most of its items have been proven useful in practice [2, 3]. Another positive effect of the ongoing use of scales from the first COPSOQ version in Germany is the generation of a database out of which a sample of 257,236 cases from employee surveys from 2015 to 2020 could be extracted for this exploration. The average participation rate of these surveys was with 61.4% relatively high in comparison to about 50% that could be expected for these kinds of surveys [19]. This may be a result of usually taking risk assessment as part of work and promoting COPSOQ as an effective instrument, being worth the 20 min (median time) to fill in the questionnaire.

The sample was not random, coming from surveys on risk-assessment in companies. But due to its size and breadth, it represented all occupational areas in Germany well according to the official statistics. Even if some items and scales entered the questionnaire at a later point in time, statistical analysis could be conducted with about 250,000 cases for 25 of the 31 scales and with a minimum of 134,896 cases in listwise perspective, i. e. including all 31 scales at the same time without asking for the reason why an answer was missing. In the perspective of single scales, the average rate of missing values was with 3.6% higher than it was in 2005 with less than 2%. Both values are acceptable in comparison with similar studies [1].

It can be noted that the pattern of mean values for all pursued scales in COPSOQ III are very similar to 2005. For example, the “Meaning of Work” has with 74.4 points now and 77.6 points in 2005 a relatively high mean value due to a distinct ceiling effect, while “Unfair Treatment” shows a relatively low mean with 21.3 and 15.4 points, respectively, showing an obvious floor effect. Technically, these effects can be tolerated, as there is still space left for variance. Also, the content seems valid, suggesting that most respondents seemed to think positively of their work, and a majority also seems to have never felt unjustly criticised, bullied or shown up in front of others. It is better to accept values this way than to abolish proven scales for an – often unreachable – ideal of statistical conformity and lose content validity by eliminating important aspects. In general, the mean values of scales kept from the first COPSOQ version as well as the scales that had been integrated in the later years ranged in an interval of 20–77 points. This is similar to 2005, when it was 15–78 points. With standard deviations, these ranged within an interval of 16.9–28.4 points now, and was 14.4–30.2 points in 2005. It can be summed up that sensitivity, variance, and distribution characteristics seem to be of the same (good) quality in COPSOQ III [2].

Some scales with remarkably low or high α that had been already part of the first version, have almost identical values. For “Feedback”, α was at both times 0.58. For “Quality of Leadership” it was 0.91 in COPSOQ III, while it was 0.89 in 2005. These scales have not been changed since 2005. “Work Privacy Conflicts” was α = 0.92 at both points in time, but it has to be noted that only two items have been kept from the old version. This means that the new items chosen for COPSOQ III by the international network as core items, are fitting well. It is different for “Degrees of Freedom”: with the reduction to two items, the scale’s α has decreased from 0.78 to 0.53. This has to be observed in future studies, but in total it seems more important that 20 out of 25 multi-item scales exceeded Cronbach’s α of at least 0.7 in COPSOQ III, while 13 even reached an α of 0.8 and more. Some scales that had an satisfying α in 2005 have increased values, like “Hiding Emotions” with α = 0.80 vs. 0.65 (2 instead of 3 items) and “Influence at Work” with α = 0.75 vs. 0.64 (unchanged since 2005) [2].

A low Cronbach’s α does not necessarily invalidate a scale. It can also be an indicator that a construct’s items describe rather different perspectives. A closer look at the scale “Degrees of Freedom” may illustrate how low values in internal consistency can be tolerated. On the one hand, the freedom to have a break and the freedom to take holidays refer to pretty different periods of time (a working day vs. a whole year), and might be very different from job to job. On the other hand, both examine the degrees of freedom to organise one’s own time. From this point of view, they describe the same thing, but the correlation of the two items (and scale reliability) can be low if only one of the two is achieved. It is the other way round with “Quality of Leadership” and “Work Privacy Conflicts” that tended in the opposite direction with their α. Exceeding a value of 0.9 seems might indicate a possible redundancy of some items’ content and the possibility to economize the scale.

Internal validity and distinctiveness have been analysed by bivariate correlation and EFA. With less than 5% of correlations showing a Pearson’s r > 0.49, the scales of the German COPSOQ III are not tied strongly to each other. Only half of the strong correlations occurred between work factors and effects, thus between causes and effects, but not within the fields of causes or effects, which would have been an indicator for possible redundancy. In 2005 the focus was not so much on the total matrix, but on correlations between scales inside the dimensions of “Demands, Influence and Possibilities for Development” and “Social Relations and Leadership”. All scales that are now part of COPSOQ III had then shown low or medium correlations except “Meaning of Work” with “Commitment to Workplace” (r = 0.52), “Support at Work” with “Sense of Community” (r = 0.52) and “Support at Work” with “Quality of Leadership” (r = 0.62) [2]. In COPSOQ III these correlations are lower but “Hiding Emotions” with “Emotional Demands” and “Work Privacy Conflicts” with Quantitative Demands” had a correlation of r = 0.53 in both cases.

Far more important is, what happened in the dimension of “Social Relations” and “Leadership” with some scales added until 2017. There seems to be a handful of new scales correlating relatively strong with each other. “Quality of Leadership” is associated with “Support at Work” (*r* = 0.60), “Trust and Justice” (*r* = 0.59), “Predictability of Work” (*r* = 0.58), and “Recognition” (*r* = 0.54). “Recognition” is connected to “Trust and Justice” (*r* = 0.61) and “Predictability of Work” (*r* = 0.51). Also, “Predictability of Work” and “Trust and Justice” are correlating (*r* = 0.58).

The reason for this is probably that some items of different scales address the same content. The question, if one is well-informed in advance concerning important decisions, changes, or plans for the future could, e. g., be understood as something a superior is responsible for. In the same way, the question, to what extent the immediate superior is good at work planning could be understood as something influencing one’s knowledge about what needs to be done. It is a deliberate decision to accentuate predictability in one case and leadership in the other by a combining them with the appropriate items. These correlations are not disquieting as far as the scales are not identical, but when it comes to multivariate analysis, they should be taken into consideration.

The noteworthy complex persisted in the EFA on work factors (Table 3), when the component “Social Relations and Leadership”, overlapped in some aspects with the component “Influence and Possibilities for Development”. The other three out of the total five components showed less overlapping. “Demands” and “Additional Factors” were especially homogenous in their content. More difficult to handle is the component combining “Quantity of Social Relations” and “Degrees of Freedom” (Breaks / Holidays) which seems vague. But instead of speculating, it seems more appropriate to accept some contingency than to demand determination here. It should be recalled that COPSOQ is itself neither a theoretical approach nor is it dedicated to a particular theoretical model. Thus, the structural congruence with a priori dimensions in Fig. 1, which goes back to the validation of a first COPSOQ version in Germany about 15 years ago [3], should be emphasised.

### The conceptual perspective

The claim of any COPSOQ questionnaire is to include aspects from a multitude of relevant psychological theories and models and not to follow one specific theory. Therefore, COPSOQ III items and scales should be beneficial for different theoretical approaches. It is not difficult to recognise in the a priori dimensions shown in Fig. 1 the order of the seminal Demand-Control model (DC) [12] complemented later with the dimension of Social support factors (DCS) [13]. But also central elements of ERI (Effort-Reward-Imbalance) or the later developed and today very popular Job Demands-Resources model (JD-R) [20] are included in COPSOQ.

The EFA on effects discriminated between a sphere of health state but also for a sphere of satisfaction, as was true already for the 2005 German version (Table 4). The new aspect of “Work Engagement” was adapted from the Utrecht Work Engagement Scale (UWES) [21] and located in the German COPSOQ III as an effect of other work factors. With this aspect, according to the JD-R model a path can be reconstructed, that unlike “Burnout Symptoms”, should influence job performance in a positive manner. The results of linear regressions in Table 5 confirmed the concept of the JD-R model, having selected influencing factors as predictors of specific dimensions, e. g., “Meaning of Work” for “Work Engagement” on one side, and “Work Privacy Conflicts” for “Burnout Symptoms” on the other side. In this context, it is a bit surprising that “Emotional Demands” played a minor role among the influencing factors in the regression model of “Burnout Symptoms” now. This contradicts some theories and the study results from 2005. This is probably a side-effect of adding the question if one has “to deal with other people’s personal problems” as an international core item to this scale in COPSOQ III. By representing a highly selective experience of certain occupational groups, it could lead to a polarisation of variance and as such to a neutralisation of the scale in some aspects. In this respect, “Hiding Emotions”, a scale with somehow substitutional content seems to have entered the group of top predictors in the regression model.

A comparison with the widely-used Effort-Reward-Imbalance model (ERI) [22] was done on the basis of the first German questionnaire. Nübling et al. used both ERI and COPSOQ together in a population-based study with the ERI-model’s original items, finding both suitable for successfully detecting risk factors for health and satisfaction outcomes [23]. There is not much reason, why this should be different with the new questionnaire version, since its relevant content was kept almost unchanged in COPSOQ III.

In a more general perspective, it could be asked why regressions on satisfaction outcomes tend to higher degrees of explained variance than those on health outcomes. Higher complexity of multi-item-scales compared to single-item-constructs as outcomes may be a reason for this ranking, but content may be the another. It could be expected that, e. g., “Job Satisfaction” is more related to workplace factors than “General Health”, which to a great extent is affected by aspects outside the sphere of work and private life.

Certainly, the distinctions between a health sphere and a satisfaction sphere, and between either cause or effect are easy to comprehend. But they are simplifications of the for more complex reality. As an example, high quantitative demands can lead to work privacy conflicts that could cause both, bad health and low satisfaction. Correlation tables (Additional file 1) and regression models (Table 5) give reasons for a more sophisticated analysis. Beyond the basic approach, statistical analyses like confirmative factor analysis (CFA) and structural equation modelling (SEM) to test models, or multilevel analysis to explore group-related variance are preferable for the future.

Meanwhile, there are a huge number of studies listed on the international network’s website combining parts of different COPSOQ versions with items and scales from other origins (www.copsoq-network.org). These cannot all be discussed. An example for critical reflexion of the old German version with COPSOQ III is Wagner et al. who have converted values of 16 scales added in 2015 with the old COPSOQ version to COPSOQ III to see if this would lead to identical results. Investigating a total sample of 948 nurses and physicians they concluded that the conversion was appropriate and useful to reveal implications for the improvement of working conditions [24].

Another example could be the study of Kuczynski et al. who have extended the Short questionnaire for Work Analysis (KFZA), which is quite popular in Germany for risk assessment, with some items that were part of the old German COPSOQ questionnaire and are also part of COPSOQ III. In the first step they used the data of 1151 employees that they had surveyed from 15 companies in 2016/2017 to test the internal validity of the scales in their newly built questionnaire. In the second step they took the longitudinal data of 293 employees in 2018 to check discriminant validity. The statistical results were satisfactory, providing incremental validity above existing instruments [25]. In this case, testing was not only motivated by cognitive interest. It aimed to comply with some recommendations of the Joint German Occupational Safety and Health Strategy (GDA) [26], too. The GDA is an initiative of government and accident insurances to support companies in carrying out psychosocial risk assessments. In a manual, work content, work organisation, social relations, physical working conditions, and new forms of work are named as typical sources of risks. The content of German COPSOQ III fits in very well, especially since scales on dissolution and on physical demands have been added [27].

However, it helps to recall the fact that the COPSOQ international network promotes the practical usability of the questionnaire. Developed as a means to improve working conditions, results should be addressable to working people. In an abstract meaning of diagnosticity, this was exemplified by pointing out the association of “Emotional Demands” with occupational areas that do not seem to exist for “Quality of Leadership” in Fig. 2. It is hardly surprising that the level of “Emotional Demands” depends on occupation. The work of e. g., medical doctors and nursing staff, social workers or teachers differs from that of farmers, technicians, engineers, or scientists in a very important aspect: they are dealing predominantly with human beings, which is known to be emotionally more demanding than dealing mostly with things. It the case of “Quality of Leadership”, it seems more difficult to imagine a similar effect caused by occupational group. Why getting along in an atmosphere of mutual trust and an efficient hierarchical relation should depend on professional activities is just not conceivable.

It is obvious, that a few psychosocial work factors can be weakly connected to occupations [28]. “Emotional Demands” and “Working environment / Physical Demands” are probably the most applicable, standing for primarily dealing with human beings and performing typically blue-collar work, respectively [27]. This makes any job exposure matrix (JEM) to predict psychosocial working conditions by knowing a person’s profession unrealistic. But what alternative is there to explain variance? It is probable that “Quality of Leadership”, like other qualities of social relationships are shaped less by abstract clusters of persons (i. e. job titles, professions) than by the those working with each other (i. e. departments, teams) [28]. This could be investigated using a multilevel analysis that would include companies’ organisational groups, such as their departments, teams, or any other relevant units.

## Conclusions

From its beginning, COPSOQ was developed for common and universal use. The first German COPSOQ version of 2005 was the result of testing the initial Danish COPSOQ I while adding some aspects of national relevance. Germany was not the only country where COPSOQ I was tested and adapted. When COPSOQ II was introduced in 2010, this was more an international than only a Danish or European event. “COPSOQ” had become a global brand over the years and is used today in a growing number of countries all over the world. Besides validation in some European countries, COPSOQ II was validated, e. g., for Malaysia [29], Canada [30], Iran [31], and Chile [32]. These studies were different in their methodology, but even more heterogenous is the large number of studies using parts of any version of the COPSOQ questionnaire in order to examine theoretical concepts and / or working conditions of selected populations for scientific reasons or for the purpose of risk assessment (www.copsoq-network.org).

The German validation of COPSOQ III is neither based on a highly selective group nor on a random sample. The sample is, with more than 250,000 cases, comparatively very large and covers all occupational areas. This is surely because Germany is one of the countries where COPSOQ is frequently used in employee surveys and data is collected anonymously in a central database. The questionnaire goes into detail but is not too long, it is based on complex theories but easy to understand and, it is one of the recommended instruments [33] for risk assessment that is required by law of German employers [34]. The results of systematic data analysis on the basis of DIN EN ISO 10075-3 suggest the same good qualities for COPSOQ III as for the first German COPSOQ version of 2005.

The FFAW, as the German group for COPSOQ-related issues, was one of the groups from six countries that took part in the testing of COPSOQ III and in publishing the results when the questionnaire was launched by the COPSOQ international network in 2019 [1]. Next to this kind of international validation, national validation studies should follow the guidelines of COPSOQ III to require a combination of the international core items with country specific items. Today there are such studies from Sweden [35], Turkey [36], Spain [37], and Germany (this article). These studies report good statistical qualities, but their results cannot be directly compared as international core items are combined with some country-specific items on scale level. It is a future task to compare the results on core item level and to find out whether differences in item values are due to methodological, cultural, or other reasons.

Following the conceptual spirit of the international network, national validation studies should be followed by studies conducted in cooperation between the affiliated national groups using the common core items. In this perspective, international comparison could mean investigating what truly global psychosocial work factors are, in contrast to influences of the local cultures. Even unique theoretical models may emerge due to the special design of COPSOQ III. Surely, all this will take a bit of time, but by now we are ready to start.

## Supplementary Information

**Additional file 1.** Origins, years of entry, affiliations and variable names of items.

**Additional file 2.** Bivariate correlations between scales.

## Data Availability

The datasets analysed during the current study are not publicly available due to dataprotection contracts with companies.
