# Supplementary material for: COPSOQ III in Germany: validation of a standard instrument to measure psychosocial factors at work
Source: J Occup Med Toxicol. 2021 Nov 16;16:50. doi: 10.1186/s12995-021-00331-1 (PMC8594291; doi:10.1186/s12995-021-00331-1)
Supplement: Supplementary file 1 — Additional file 1. Origins, years of entry, affiliations and variable names of items. [file 12995_2021_331_MOESM1_ESM.pdf]

Additional file 1: Origins, years of entry, affiliations and variable names of items

**Scale**	**Item**	**International Core/*Additional item name**	**Item name in German version**	**Origin (see below)**	**Year of entry in German version**
Quantitative Demands	Do you have to work very fast?	WP1	B1_1	1	2005
	Do you work at a high pace throughout the day?	WP2	B1_2	2	2017
	How often do you not have time to complete all your work tasks?	QD4	B1_3	1	2005
	Do you get behind with your work?	QD3	B1_4	1	2017
	Do you have to do overtime?	*CT5	B1_5	1	2005
Emotional Demands	Do you have to deal with other people’s personal problems as part of your work?	EDX2	B1_6	2	2017
	Is your work emotionally demanding?	ED3	B1_7	1	2005
Hiding Emotions	Does your work require that you hide your feelings?	*HE2	B1_8	1	2005
	Does your work require that you do not state your opinion?	*HE4	B1_9	1	2005
Work Privacy Conflicts	The demands of my work interfere with my private and family life.	-	B2_1	3	2005
	Due to work-related duties, I have to make changes to my plans for private or family activities.	-	B2_2	3	2005
	My work drains so much of my energy that it has a negative effect on my private life.	WF2	B2_3	2	2017
	My work takes so much of my time that it has a negative effect on my private life.	WF3	B2_4	2	2017
Dissolution	I take care of work-related tasks outside of my working time as well.	-	B2_5	4	2017
	I’m available in my free time for people with whom I deal professionally.	-	B2_6	4	2017
Influence at Work	Do you have a large degree of influence on the decisions concerning your work?	INX1	B3_1	1	2005
	Can you influence the amount of work assigned to you?	*IN3	B3_2	1	2005
	Do you have any influence on what you do at work?	*IN4	B3_3	1	2005
Degrees of Freedom (Breaks / Holidays)	Can you decide when to take a break?	*CT1	B3_4	1	2005
	Can you take holidays more or less when you wish?	*CT2	B3_5	1	2005
Possibilities for Development	Is your work varied?	*VA1	B4_1	1	2005
	Do you have the possibility of learning new things through your work?	PD2	B5_1	1	2005

	Can you use your skills or expertise in your work?	PD3	B5_2	1	2005
Meaning of Work	Is your work meaningful?	MW1	B5_3	1	2005
	Do you feel that the work you do is important?	*MW2	B5_4	1	2005
Commitment to Workplace	Are you proud of being part of this company?	*CW5	B5_5	1	2005
	Do you enjoy telling others about your place of work?	*CW1	B5_6	1	2005
Predictability of Work	At your place of work, are you informed well in advance concerning for example important decisions, changes, or plans for the future?	PR1	B6_1	1	2005
	Do you receive all the information you need in order to do your work well?	PR2	B6_2	1	2005
Role Clarity	Does your work have clear objectives?	CL1	B6_3	1	2005
	Do you know exactly which areas are your responsibility?	CL2	B6_4	1	2005
	Do you know exactly what is expected of you at work?	CL3	B6_5	1	2005
Role Conflicts	Are contradictory demands placed on you at work?	CO2	B6_6	1	2005
	Do you sometimes have to do things, which ought to have been done in a different way?	CO3	B6_7	1	2005
	Do you sometimes have to do things, which seem to be unnecessary?	*IT1	B6_8	1	2005
Quality of Leadership	To what extent would you say that your immediate superior makes sure that the members of staff have good development opportunities?	*QLX1	B7_1	1	2005
	To what extent would you say that your immediate superior gives high priority to job satisfaction?	*QL2	B7_2	1	2005
	To what extent would you say that your immediate superior is good at work planning?	QL3	B7_3	1	2005
	To what extent would you say that your immediate superior is good at solving conflicts?	QL4	B7_4	1	2005
Support at Work	How often do you get help and support from your colleagues, if needed?	SCX1	B8_1	1	2005
	How often are your colleagues willing to listen to your problems at work, if needed?	*SCX2	B8_2	1	2005
	How often do you get help and support from your immediate superior, if needed?	SSX2	B8_3	1	2005
	How often is your immediate superior willing to listen to your problems at work, if needed?	*SSX1	B8_4	1	2005
Feedback	How often does your immediate superior talk with you about how well you carry out your work?	*SSX3	B8_5	1	2005
	How often do your colleagues talk with you about how well you carry out your work?	*SC3	B8_6	1	2005
Quantity of Social Relations	Is it possible for you to talk to your colleagues while you are working?	-	B8_7	1	2005
Sense of Community	Is there a good atmosphere between you and your colleagues?	SW1	B8_8	1	2005
	Is there good co-operation between the colleagues at work?	*SW2	B8_9	1	2005

Unfair Treatment	How often do you feel unjustly criticised, bullied or shown up in front of others by your colleagues or your superior?	-	B8_1	5	2005
Trust and Justice	Does the management trust the employees to do their work well?	TM1	B8a_1	2	2010
	Can the employees trust the information that comes from the management?	TMX2	B8a_2	2	2010
	Are conflicts resolved in a fair way?	JU1	B8a_3	2	2010
	Is the work distributed fairly?	JU4	B8a_4	2	2010
Recognition	Is your work recognized and appreciated by the management?	*JU2	B8a_5	2	2017
Work Environment / Phys. Demands	How often do you have to do physically strenuous work such as lift, carry or raise heavy objects?	-	B8b_1	6	2014
	How often are you exposed to noise or loud background noise at your workplace?	-	B8b_2	6	2014
	How often do you come in contact with chemicals or hazardous substances at your work?	-	B8b_3	6	2014
	How often are you exposed to extreme temperatures or a draft at your workplace?	-	B8b_4	6	2014
	How often are you exposed to poor air quality at work, e.g. cigarette smoke, gases or similar?	-	B8b_5	6	2014
	How often are you exposed to poor lighting conditions at work, for example, glaring or low light?	-	B8b_6	6	2014
Job Insecurity	Are you worried about becoming unemployed?	JI1	B9_1	1	2005
	Are you worried about new technology making you redundant?	*JI2	B9_2	1	2005
	Are you worried about it being difficult for you to find another job if you became unemployed?	JI3	B9_3	1	2005
Insecurity over Working Conditions	Are you worried about being transferred to another job against your will?	IW1	B9_4	1	2005
	Are you worried about the timetable being changed (shift, weekdays, time to enter and leave, ...) against your will?	*IW3	B9_5	Int. Network	2017
	Are you worried about a decrease in your salary?	*IW4	B9_6	Int. Network	2017
Intention to leave Profession / Job	In the past 12 months, how often have you thought about giving up your profession?	-	B10_1	7	2005
	In the past 12 months, how often have you thought about changing your job?	-	B10_2	FFAW	2005
Job Satisfaction	Regarding your work in general. How pleased are you with your work prospects?	*JS1	B11_1	1	2005
	Regarding your work in general. How pleased are you with the people you work with?	-	B11_2	1	2005
	Regarding your work in general. How pleased are you with the physical working conditions?	*JS2	B11_3	1	2005
	Regarding your work in general. How pleased are you with the way your group is run?	-	B11_4	1	2005
	Regarding your work in general. How pleased are you with the way your abilities are used?	*JS3	B11_5	1	2005
	Regarding your work in general. How pleased are you with your salary?	*JS5	B11_6	Int. Network	2017

	Regarding your work in general. How pleased are you with your job as a whole, everything taken into consideration?	JS4	B11_7	1	2005
Work Engagement	At my work, I am full of energy.	*WE1	B14_1	8	2017
	I am enthusiastic about my job.	*WE2	B14_2	8	2017
	I am immersed in my work.	*WE3	B14_3	8	2017
General Health	Your present health state if you evaluate the best conceivable state of health at 10 points and the worst at 0 points.	*GH2	B12	1	2005
Burnout Symptoms	How often do you feel physically exhausted?	*BO2	B13_1	1	2005
	How often do you feel emotionally exhausted?	*BO3	B13_2	1	2005
	How often do you feel worn out?	*BO1	B13_3	1	2005
Presenteeism	How often does it occur that you come to work, even though you really feel unwell and sick?	-	B13_4	9	2010
Inability to Relax	How often are you not able to stop thinking about work in your free time?	-	B13_5	10	2017

2 COPSOQ I (Nübling, Stößel, Hasselhorn, Michaelis & Hofmann, 2005), (Kristensen, Hannerz, Høgh & Borg, 2005)

5 COPSOQ II (Pejtersen, Kristensen, Borg & Bjorner, 2010)

38 Netemeyer et al. (Netemeyer, Boles & McMurrian, 1996)

39 IGA (Hassler, Rau, Hupfeld & Paridon, 2016)

40 BIBB/IAB (Zentralarchiv für Empirische Sozialforschung, o.J.)

41 BIBB/BAuA (Fuchs, 2006)

42 NEXT (Hasselhorn et al., 2003)

21 UWES (Schaufeli, Bakker & Salanova, 2016)

43 Krause et al. (Krause, Dorsemagen, Stadlinger & Baeriswyl, 2012)

44 Sonnentag et al. (Sonnentag, Unger & Nägel, 2013)
