# Supplementary material for: COPSOQ III in Germany: validation of a standard instrument to measure psychosocial factors at work
Source: J Occup Med Toxicol. 2021 Nov 16;16:50. doi: 10.1186/s12995-021-00331-1 (PMC8594291; doi:10.1186/s12995-021-00331-1)
Supplement: Supplementary file 2 — Additional file 2. Bivariate correlations between scales. [file 12995_2021_331_MOESM2_ESM.pdf]

Additional file 2: Bivariate correlations between scales

Pearson correlation (r)*	Demands					Influence and possibilities for development					Social relations and leadership (part 1)					
	Quanti-tative Demands	Emotio-nal Demands	Hiding Emotions	Work Privacy Con-flicts	Dis-solution	Influ-ence at Work	Degrees of Freedom (Breaks / Holidays)	Possi-bilities for Develop-ment	Mea-ning of Work	Commit-ment to Work-place	Predict-ability of Work	Role Clarity	Role Conflicts	Quality of Leadership	Support at Work	Feed-back
Quantitative Demands	--															
Emotional Demands	0.39	--														
Hiding Emotions	0.36	0.53	--													
Work Privacy Conflicts	0.53	0.41	0.45	--												
Dissolution	0.34	0.34	0.22	0.39	--											
Influence at Work	-0.03	0.12	-0.15	-0.12	0.18	--										
Degrees of Freedom (Breaks / Holidays)	-0.11	-0.11	-0.19	-0.23	-0.06	0.35	--									
Possibilities for Development	0.12	0.20	-0.07	-0.04	0.21	0.47	0.28	--								
Meaning of Work	-0.03	0.07	-0.16	-0.15	0.05	0.25	0.07	0.45	--							
Commitment to Workplace	-0.14	-0.06	-0.27	-0.27	0.08	0.29	0.15	0.40	0.49	--						
Predictability of Work	-0.20	-0.11	-0.30	-0.29	-0.04	0.30	0.17	0.31	0.33	0.44	--					
Role Clarity	-0.12	-0.07	-0.19	-0.20	-0.07	0.16	0.05	0.21	0.43	0.34	0.49	--				
Role Conflicts	0.36	0.30	0.42	0.42	0.22	-0.14	-0.15	-0.11	-0.26	-0.31	-0.44	-0.32	--			
Quality of Leadership	-0.19	-0.10	-0.28	-0.29	-0.03	0.29	0.20	0.35	0.30	0.45	0.58	0.39	-0.39	--		
Support at Work	-0.17	-0.10	-0.26	-0.29	-0.05	0.25	0.25	0.33	0.25	0.37	0.45	0.32	-0.31	0.62	--	
Feedback	-0.05	-0.01	-0.15	-0.13	0.05	0.22	0.09	0.26	0.20	0.31	0.34	0.25	-0.13	0.44	0.45	--

(continued)

Pearson correlation (r)*	Demands					Influence and possibilities for development					Social relations and leadership (part 1)					
	Quantitative Demands	Emotional Demands	Hiding Emotions	Work Privacy Conflicts	Dissolution	Influence at Work	Degrees of Freedom (Breaks / Holidays)	Possibilities for Development	Meaning of Work	Commitment to Workplace	Predictability of Work	Role Clarity	Role Conflicts	Quality of Leadership	Support at Work	Feedback
Quantity of Social Relations	-0.13	-0.10	-0.16	-0.16	0.00	0.17	0.27	0.15	0.05	0.16	0.12	0.05	-0.05	0.14	0.28	0.20
Sense of Community	-0.09	-0.09	-0.21	-0.21	0.00	0.16	0.17	0.24	0.20	0.28	0.28	0.23	-0.23	0.33	0.49	0.30
Unfair Treatment	0.16	0.12	0.28	0.27	0.08	-0.20	-0.22	-0.23	-0.19	-0.24	-0.31	-0.22	0.36	-0.39	-0.42	-0.15
Trust and Justice	-0.23	-0.16	-0.32	-0.34	-0.06	0.25	0.18	0.29	0.35	0.48	0.58	0.43	-0.48	0.59	0.49	0.31
Recognition	-0.17	-0.07	-0.26	-0.28	0.00	0.29	0.13	0.32	0.31	0.45	0.51	0.35	-0.37	0.54	0.41	0.40
Work Environment / Phys. Demands	0.07	0.06	0.14	0.22	0.03	-0.19	-0.34	-0.23	-0.04	-0.14	-0.21	-0.01	0.24	-0.22	-0.23	-0.05
Job Insecurity	0.00	-0.07	0.08	0.09	-0.06	-0.20	-0.15	-0.25	-0.13	-0.10	-0.15	-0.08	0.09	-0.14	-0.18	-0.06
Insecurity over Working Conditions	0.11	0.05	0.20	0.26	0.03	-0.23	-0.28	-0.24	-0.14	-0.19	-0.27	-0.14	0.26	-0.24	-0.26	-0.08
Intention to leave Profession / Job	0.24	0.16	0.30	0.39	0.13	-0.18	-0.12	-0.25	-0.35	-0.44	-0.32	-0.28	0.35	-0.34	-0.28	-0.17
Job Satisfaction	-0.25	-0.15	-0.37	-0.42	-0.06	0.35	0.28	0.44	0.41	0.56	0.55	0.40	-0.47	0.64	0.54	0.36
Work Engagement	-0.09	-0.01	-0.21	-0.29	0.06	0.29	0.10	0.43	0.51	0.56	0.37	0.37	-0.31	0.38	0.30	0.28
General Health	-0.18	-0.15	-0.24	-0.33	-0.07	0.17	0.18	0.20	0.17	0.28	0.25	0.16	-0.24	0.26	0.29	0.15
Burnout Symptoms	0.39	0.31	0.39	0.53	0.19	-0.18	-0.21	-0.17	-0.20	-0.31	-0.31	-0.21	0.38	-0.31	-0.28	-0.16
Presenteeism	0.26	0.18	0.26	0.32	0.20	-0.15	-0.21	-0.14	-0.09	-0.17	-0.22	-0.10	0.27	-0.21	-0.22	-0.09
Inability to Relax	0.29	0.21	0.23	0.37	0.29	-0.02	-0.08	0.01	-0.06	-0.11	-0.16	-0.13	0.23	-0.17	-0.18	-0.07

(continued)

Pearson Correlation (r)*	Social relations and leadership (part 2)					Additional factors			Effects						
	Quantity of Social Relations	Sense of Com-munity	Unfair Treatment	Trust and Justice	Recog-nition	Work Environment / Phys. Demands	Job Inse-curity	Insecurity over Working Conditions	Intention to leave Profession / Job	Job Satis-faction	Work Engage-ment	General Health	Burnout Symptoms	Presente-eism	Inability to Relax
Quantity of Social Relations	--														
Sense of Community	0.23	--													
Unfair Treatment	-0.10	-0.39	--												
Trust and Justice	0.13	0.35	-0.37	--											
Recognition	0.11	0.25	-0.31	0.63	--										
Work Environment / Phys. Demands	-0.08	-0.20	0.28	-0.24	-0.21	--									
Job Insecurity	-0.09	-0.14	0.20	-0.17	-0.17	0.21	--								
Insecurity over Working Conditions	-0.12	-0.19	0.30	-0.30	-0.26	0.37	0.56	--							
Intention to leave Profession / Job	-0.06	-0.23	0.30	-0.37	-0.34	0.12	0.09	0.21	--						
Job Satisfaction	0.18	0.44	-0.44	0.61	0.57	-0.36	-0.19	-0.37	-0.54	--					
Work Engagement	0.07	0.28	-0.24	0.41	0.40	-0.10	-0.13	-0.17	-0.47	0.54	--				
General Health	0.14	0.23	-0.26	0.29	0.25	-0.25	-0.21	-0.26	-0.28	0.40	0.32	--			
Burnout Symptoms	-0.14	-0.25	0.32	-0.35	-0.31	0.26	0.18	0.29	0.41	-0.48	-0.39	-0.54	--		
Presenteeism	-0.11	-0.17	0.26	-0.24	-0.20	0.26	0.17	0.28	0.25	-0.33	-0.13	-0.37	0.47	--	
Inability to Relax	-0.09	-0.14	0.18	-0.19	-0.14	0.09	0.11	0.16	0.22	-0.22	-0.11	-0.24	0.37	0.30	--

* Statistical significance is for correlations $p < 0.01$.
